# Some Nutritional Value Aspects of Barley and Oat and Their Impact in Human Nutrition and Healthy Life

**DOI:** 10.3390/plants13192764

**Published:** 2024-10-02

**Authors:** Diana Camelia Batîr Rusu, Danela Murariu, Roxana Gheorghita, Mariana Graur

**Affiliations:** 1 Suceava Genebank, 1 Mai Blvd., No. 17, 720224 Suceava, Romania; diviana.diana@gmail.com (D.C.B.R.); danela.murariu@svgenebank.ro (D.M.); 2 Department of Biological and Morphofunctional Sciences, College of Medicine and Biological Sciences, Stefan cel Mare University of Suceava, University 13, 720229 Suceava, Romania; 3 Department of Medical-Surgical and Complementary Sciences, College of Medicine and Biological Sciences, Stefan cel Mare University of Suceava, University 13, 720229 Suceava, Romania; graur.mariana@gmail.com

**Keywords:** germplasm, diversity, nutritional properties, genotypes, landraces, functional food

## Abstract

Nowadays, there is a general concern regarding the increasing global talk about functional foods that respond to our demands and needs as consumers in order to maintain health and body weight through a correctly balanced diet. Cereals are key elements of nutrition and a healthy diet, and they also play a significant role in health promotion due to the useful nutrient content. Therefore, this work aims to identify barley and oat genotypes suitable for human nutrition and to achieve practical results for their widespread use in preventing or treating certain chronic diseases by analyzing the nutritional and physical properties of 52 genotypes of oat and barley conserved in Suceava Gene Bank, Romania. The first part of this manuscript is the presentation of these accessions and the evaluation of their most important properties. For oat and barley cultivars, detailed processing was carried out, involving the computation of variation amplitude, coefficients of correlation and cluster analyses, both for biochemical (protein, lysine and tryptophan contents) and physical (test weight and seed weight) properties. The results indicated high variability between oat and barley varieties. Thus, according to the results, the 26 varieties of oat exhibited almost double the content of lysine compared to barley seeds, while tryptophan had higher values in barley than in oat seeds. Overall, both species play an essential role in human nutrition, barley being important because of its high protein content and higher productivity compared to oats, which, although not as productive, have better quality seeds due to their higher lysine content. The results presented are not only of scientific interest but also have practical implications for agriculture, food safety, nutrition and human health. The documented information will facilitate new studies needed to contribute to improving human nutrition and health.

## 1. Introduction

In modern society, the food industry is looking towards the production of functional cereal-based foods due to consumers’ tendencies towards a healthier and more nutritionally appropriate diet [1,2]. Oat and barley are used for human consumption in a very small percentage of the population, although they have valuable biochemical attributes, especially a high protein content, whose nutritional quality is defined by the composition of some essential amino acids (lysine and tryptophan) [3,4,5].

A plant-based diet includes cereals, fruits and vegetables that are rich in nutritious carbohydrates, fats, vitamins, minerals, fiber and other phytonutrients. Antioxidant substances, including vitamin C, selenium and vitamin E, are abundant in plant-based diets, helping the body reduce oxidative stress, which lowers the risk of disease and promotes a healthy life [6].

Nowadays, the concept of therapeutic use of plants is not sufficiently developed. Two thousand five hundred years ago, Hippocrates accepted the idea, “Let food be thy medicine and medicine be thy food” [7]. However, with the development of modern drug therapy in the 19th century, this “food as medicine” theory was forgotten. The importance of nutrition in disease prevention and health promotion was highlighted again in the 1900s. Throughout time, scientists have always been concerned with identifying vitamins and their importance in the prevention of various disorders caused by dietary deficiencies [8,9,10].

Some factors, including urbanization and its effects, demographic change and population aging, food security, the loss of traditional food culture and awareness of deteriorating health caused by a fast-paced lifestyle, poor choices of convenient foods and a competitive food market, have led to the development of functional foods. Also, the inappropriate level of physical activity, self-medication, a lack of information from authorities, and the relationship between diet and health contribute to an unorganized lifestyle.

A functional food may be considered a wellness promotion tool and refers to any food or food ingredient with benefits in improving health or avoiding disease risk with notable nutritional effects [11].

In many cases, healthy foods are considered functional foods because they contain biologically active components with health benefits for the prevention and management of diseases. The Food and Agriculture Organization (FAO) defines functional foods as foods that contain nutrients as well as other beneficial components with potential positive effects on health [12].

Today, these functional foods have gained the attention of researchers from many fields, such as nutrition and dietetics. Due to their antimicrobial, antioxidant and anticancer properties, these products have the potential to reduce diseases by promoting a balanced lifestyle [13,14,15].

Due to high consumer demand for increasingly healthy foods, the industry is interested in developing functional cereal-based products. Cereals such as oat and barley, rich in nutritious phytochemicals, represent an excellent base in order to develop products for various targeted consumer groups.

Cereals are cultivated for their seeds, which are important in human and animal diets, or for industrial purposes, due to their high starch and protein contents. They are the main source of carbohydrates and energy in the human diet and contribute significantly to meeting protein requirements. The main phytochemicals present in cereal grains are phenolic acids, flavones, phytic acid, ferulic acid, flavonoids, coumarins and terpenes. In addition, cereal germs contain vitamins E, B1, B2, and B3; minerals; and fiber, with a role in cancer prevention or in the management of chronic diseases such as arthritis, coronary heart disease, type-2 diabetes and osteoporosis [16,17].

Oat and barley have a significant role in the human diet, are rich in phytonutrients and represent valuable ingredients for the development of functional foods. Many studies presented the capacity of oat to reduce the risk of heart disease by lowering LDL cholesterol [18], and barley has positive physiological effects, boosts metabolism, helps lower cholesterol and controls blood glucose due to its high content of dietary fiber, especially *β* glucan [19].

The development of whole grains as functional foods for human consumption is a difficult task. However, the use of new cereal-processing technologies improves their use and health potential, as well as the consumers’ acceptance.

Taking into account the importance of functional foods in a balanced lifestyle, this study aims to identify oat and barley genotypes for human consumption due to their properties, rich bioavailability, and widespread use as functional foods.

Thus, this study analyzed the characteristics of oat and barley genotypes using biochemical (protein, lysine and tryptophan contents) and physical (test weight and seed weight) properties in order to evaluate the variability of studied accessions and compare the results based on these markers.

## 2. Materials and Methods

The samples used to evaluate the variability of biochemical and physical traits of the barley and oat genotypes consist of:

- 26 accessions of *Avena sativa* L. (oat);
- 26 accessions of *Hordeum vulgare* L. var. *distichon* Alef. (spring barley).

The two species mentioned above present the following biological statuses:

- *Avena sativa* L.
  - local landraces—22;
  - inbred lines—4.
- *Hordeum vulgare* L. var. distichon Alef.
  - local landraces—24;
  - inbred lines—1;
  - obsolete cultivars—1.

**Figure 1:**
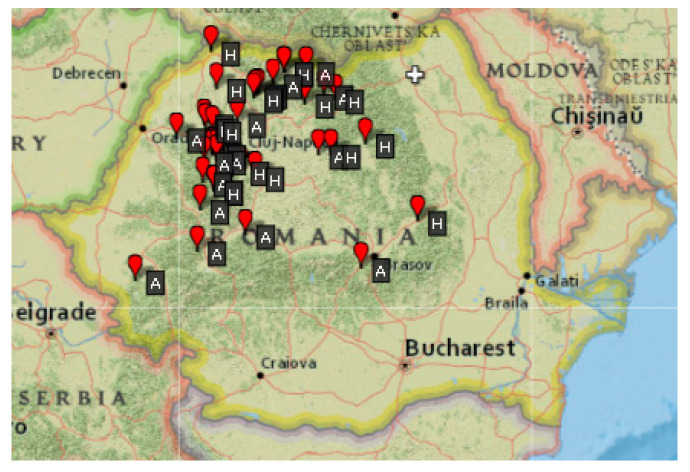
Origin of barley (H) and oat (A) local landraces.

The analyzed accessions have different origins, but all come from Romania. Some of them were collected from different sites in Romania (Figure 1, Table 1), and the others were received from Romanian breeding institutions (NARDI Fundulea, ARDS Suceava).

The seeds of 26 barley and 26 oat genotypes were analyzed in the laboratory as follows:

- Nutritional properties—protein and essential amino acid contents (lysine and tryptophan);
- Physical characteristics—test weight (kg/hL) and seed weight (g).

For the analysis of total protein and essential amino acids (lysine and tryptophan) content, different methods were used, such as micro-Kjeldahl, biuret and colorimetric evaluation (ninhydrin test, papain extraction test) and physical methods for the determination of test (TW) and seed weight (SW).

Seeds of oat and barley accessions were finely ground and stored at −20 °C until the time of testing.

Although colorimetric methods permit an easier determination of the protein content of different samples, they cannot be applied directly to cereal flours because of starch interference. Therefore, it was necessary to carry out a cereal extraction step in order to quantify the proteins. For this purpose, in order to allow the protein compounds to pass into the liquid phase, ground and defatted samples were resuspended in 1.5 mL of 0.1 n NaOH basic solution, sonicated for 30 min and centrifuged at room temperature for 15 min at 15,000 rpm.

The dry matter content was determined in a forced air circulation tube by heating at 105 °C.

The micro-Kjeldahl method and the biuret method were used to evaluate the total protein content; this method is a rapid one and uses a small number of seeds, i.e., 30–100 mg of flour. The spectrophotometer analysis was used to obtain the calibration curve necessary to calculate the extracted protein content.

The nutritional properties of oats and barley are determined by the lysine and tryptophan content of the seed and the protein quality. In addition to the content, very important is the quality of the protein, which is ensured by the ratio of essential: non-essential amino acids, of which essential amino acids must be higher.

Lysine is considered the most important essential amino acid and improves the quality of the proteins in which it is found [20]. Oat avenins proteins contain more lysine than barley hordeins, which basically have a smaller amount of lysine. Because of its low content in cereal protein and high nutritional quality, lysine is the most researched amino acid.

Due to its simplicity and reproducibility, the colorimetric method of the ninhydrin test was used for the analysis of tryptophan content. The hydrolysate obtained from the protein determined by the biuret method was treated with ninhydrin, heated at 65 °C and measured spectrophotometrically at 540 ± 10 nm. For the purpose of plotting the calibration curve, several samples with minimum, medium and maximum extinction were selected and subjected to extraction [20].

Regarding the methods used to determine physical characteristics of seeds, although cereal grains present common characteristics, they differ in certain properties that determine their specific quality. The main indicators, specific to cereal grains, are moisture content; test weight, or specific mass (kg/hL); seed weight, or relative mass (g); and shape and size of the grains.

The statistical analysis involved the correlations and corresponding regressions between the analyzed physico-chemical components. Also, the variation amplitude (min-max), variance, standard deviation and variability coefficient were calculated. Pearson correlation coefficients and Euclidean distances were analyzed using SPSS 2.0 software.

## 3. Results

The analysis was carried out based on the physico-chemical properties of the two species studied and their comparative analysis in order to identify which oat and barley genotypes possess a higher nutritional value and represent benefits for consumers’ health. The accessions taken into evaluation are presented in Table 1.

### 3.1. Physical and Some Nutritional Properties of the Oat Genotypes

In oat genotypes, after analysis of the protein, lysine and tryptophan content and physical evaluation (TW and SW) to highlight the variability of the physico-chemical properties of the oat seed accessions, the variation amplitudes for protein, lysine, tryptophan contents, test weight and seed weight were calculated (Table 2).

According to results from Table 2, the 26 studied oat accessions showed different protein concentrations, ranging from 7.02 to 14.23% d.m. The highest protein content was obtained from accession SVGB-5098, i.e., 14.23% d.m, and the lowest protein content from SVGB-14042, with a value of 7.02% d.m., which comes from Peteritea, Maramureș County. The average protein content of the 26 analyzed accessions was 10.45% d.m., which was also confirmed by Sudheesh in 2022, who stated that oat seeds can have a protein content between 9 and 20% d.m. [21].

In the case of essential amino acids content, the amplitudes of variation were much lower, with lysine content ranging from 1.35 to 3.29% of the total protein and tryptophan from 0.41 to 1.05% of the total protein.

Regarding lysine content, the maximum value was observed at SVGB-15103, originating from Păduriș, Sălaj County, with a value of 3.29% of the total protein, and minimal content (1.35% of the total protein) was obtained from accession SVGB-5535, originating from Moisei, Maramureș County. The average lysine content for our 26 analyzed samples was 2.22% of the total protein content. In 2017, Murariu and Plăcintă [22] mentioned that lysine from oat grains improved the biological value because the protein from oat seeds contains a higher amount of lysine compared to other cereals.

In terms of tryptophan content, the highest value of 1.05% of the total protein was obtained for SVGB-5655 from Râșca, Cluj County, and the lowest resulted from accession SVGB-5097, with a value of 0.41% of the total protein. The average tryptophan content of 0.75% of the total protein was found in all analyzed genotypes (*n* = 26), a lower value compared to the lysine content; the same results were mentioned by Zwer in 2017 [23], who specified that oat grains have a unique composition and a different fraction of protein distribution, with a good balance of essential amino acids content, but with a low tryptophan content.

The maximum value (47.4 kg/hL) of TW was recorded in accession SVGB-5535 from Moisei, Maramureș County, while the minimum value (31.8 kg/hL) was obtained from SVGB-5452 (Săsciori, Alba County). The average value for the analyzed oat genotypes was 37 kg/hL, in agreement with results mentioned by Murariu in 2017 [22], which showed that TW in oat falls within the range 31–50 kg/hL.

In the case of SW, the maximum value (34 g) was recorded in SVGB-15103 from Păduriș, Sălaj County, and the minimum value (14 g) was recorded in accession SVGB-5655 from Râșca, Cluj County. The average value of the SW for the 26 analyzed genotypes was 22.65 g (Table 2), which is also specified in the literature, where it is noted that SW is influenced by climatic conditions and cultivation area; for example, in Romania, this indicator varies between 14 and 36 g [22].

The coefficient of variation was determined in order to evaluate the level of variability of the biochemical and physical properties of oat accessions. According to the results presented in Table 2, the protein and tryptophan contents and the seed weight have the highest variation coefficients (16.6–21.15%).

**Figure 2:**
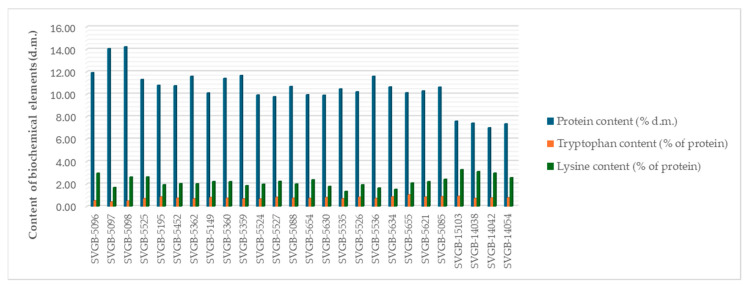
Protein, tryptophan and lysine contents of the 26 oat seed accessions.

For oat, we can observe different values between the samples regarding the protein content, but the differences are less noticeable in terms of the two essential amino acids contents (Figure 2).

**Figure 3:**
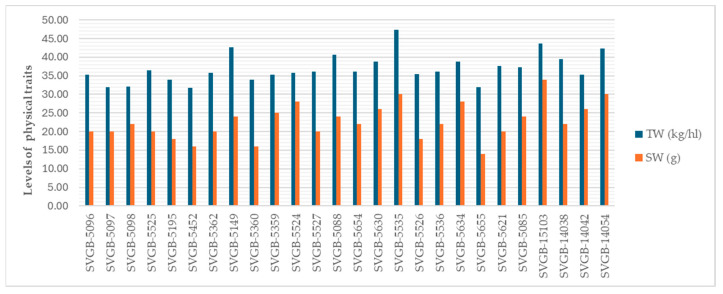
Values of TW and SW of 26 oat genotypes.

According to the results presented in Figure 3, we can observe that there is a medium to high variability of the TW and SW values.

In order to identify possible links between physico-chemical properties, Pearson correlation coefficients were calculated using SPSS 2.0 software (Table 3).

**Figure 4:**
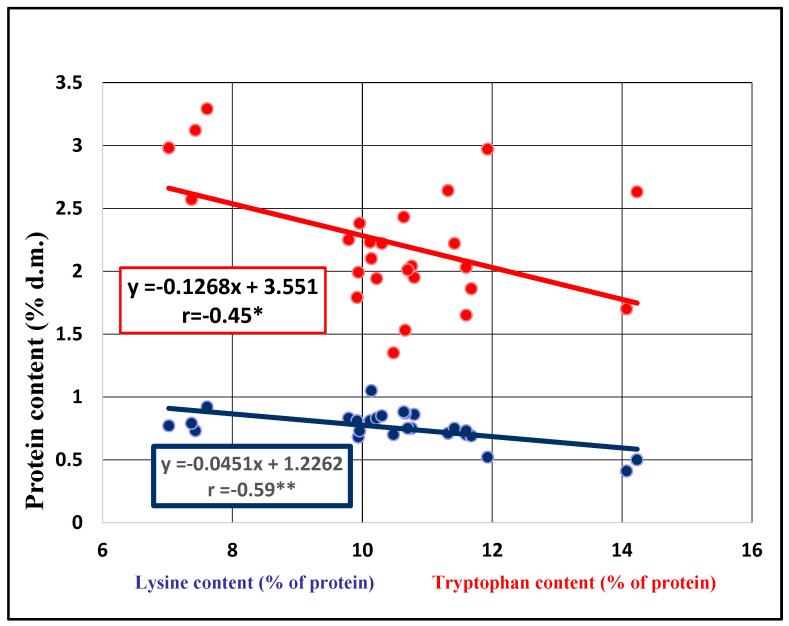
Regression lines between protein, lysine and tryptophan contents of 26 oat seed accessions.

**Figure 5:**
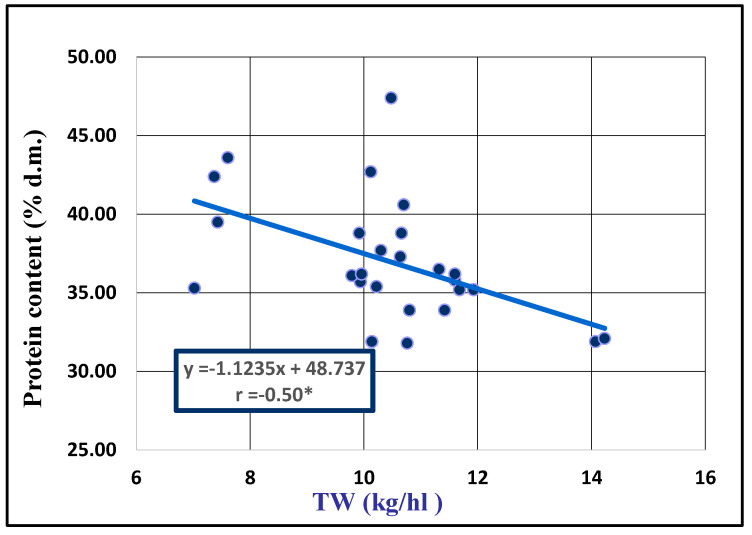
Regression line between protein content and TW of 26 oat seed accessions.

Distinctly significant negative correlations were observed both between protein and lysine contents (r = −0.591 **) and between protein content and TW (r = −0.502 **). There were also significant negative correlations between protein and tryptophan contents (r = −0.447 *) and between protein content and SW (r = −0.433 *). A highly significant positive correlation coefficient was observed between SW and TW (r = 0.747 ***). In order to highlight these correlations, we also plotted the corresponding regression lines (Figure 4 and Figure 5).

A negative relationship between protein, lysine and tryptophan contents (Figure 4), as well as between protein content and TW (Figure 5), was observed. These results showed that as protein content increases, essential amino acid content decreases. Also, an increase in kernel size does not also lead to an increase in protein content, a fact also confirmed by Redaelli in 2013 [24], even if there are less investigations in the field of protein quality in seed oat and relatively few in terms of essential amino acid composition.

### 3.2. Physical and Some Nutritional Properties of the Barley Genotypes

In the case of the barley genotypes taken in this study, the amplitude of variation between protein, lysine and tryptophan contents and test and seed weight are presented in Table 4.

According to the lab evaluation, the maximum protein content, 14.67% d.m., was observed in the accessions SVGB-10251 and SVGB-7891, originating from Agriș, Satu Mare County, and Brețcu, Covasna County, respectively. The minimum protein content (8.2% d.m.) was observed in accession SVGB-15119 from Văleni, Cluj County. The average protein content of the samples (*n* = 26) was 12.26% d.m., but the nutritional components in barley grains can be improved by selecting breeding material for the purpose of creating varieties with higher nutritional values, a fact mentioned by Russu in 2015 [25].

Regarding lysine, the maximum content (1.89% of the total protein) was recorded for accession SVGB-15119 from Văleni, Cluj county, and the minimum (0.89% of the total protein) for SVGB-7262 from Hodoșa, Harghita county. The average lysine content of analyzed genotypes was 1.48% of the total protein (Table 4). Although, in the literature, some studies by Biel in 2020 [26] mentioned that barley grains are richer in lysine content compared to other cereals’ seeds, the analyses carried out in this paper showed that barley grains have a lower lysine content compared to oat grains.

Regarding tryptophan content, the highest value (1.14% of the total protein) was noticed in accession SVGB-10192 from Săliște, Maramureș County, and the lowest (0.62% of the total protein) was obtained in SVGB-15110, from Călățele, Cluj County. The average tryptophan content (0.86% of the total protein) is a rather low value, also mentioned by Băișan in 2021 [27], who specified that prolamins represent up to 70% d.m. of the total protein in barley. Nevertheless, the composition of the two essential amino acids, lysine and tryptophan, is low.

Regarding the analyzed physical properties, the maximum value of the TW (68.9 kg/hL) was recorded in SVGB-14073 from Vima Mare, Maramureș County, while the minimum value (34.1 kg/hL) was obtained from accession SVGB-6605 (inbred line), received from the Agricultural Research and Development Station of Suceava. The average value of the TW of the barley genotypes analyzed was 62.3 kg/hL, much higher compared with the TW of oat grains, also highlighted by Murariu [22] in her studies, where she reported a TW of 58–80 kg/hL.

In the case of the SW, the maximum value (57 g) was identified in accession SVGB-15039 from Pleșca, Sălaj County, and the minimum value (30 g) was recorded in SVGB-7301 and SVGB-7262 from Hodac, Mureș County, and Hodoșa, Harghita County, respectively. The average value of the SW for the genotypes analyzed was 40.54 g, almost double than that of oat, but in the range of 23–58 g mentioned in the literature [22].

**Figure 6:**
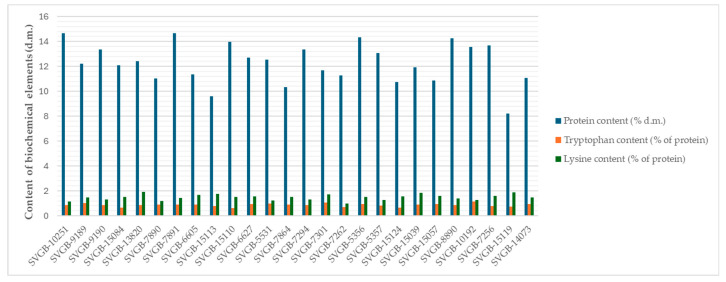
Protein, tryptophan and lysine contents of 26 barley seed accessions.

The biochemical analyses carried out on barley grains evidenced differences between accessions, especially in terms of protein content, even if they were insignificant for the two essential amino acids tested (Figure 6).

Regarding the level of variability of the physical and nutritional properties of the analyzed barley genotypes, it can be observed that, in the case of this plant species, both the quality and physical attributes show a small to middle variation (CV% = 10–16%), as shown in Table 4.

**Figure 7:**
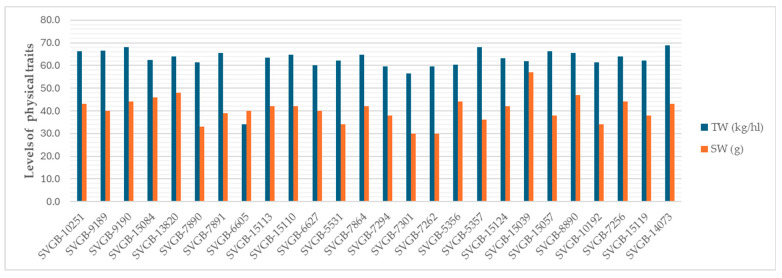
Values of TW and SW of 26 barley genotypes.

A high variability of TW and SW between barley genotypes can be observed in Figure 7.

In order to highlight the relationship between the physico-chemical properties, we calculated Pearson correlation coefficients (Table 5).

**Figure 8:**
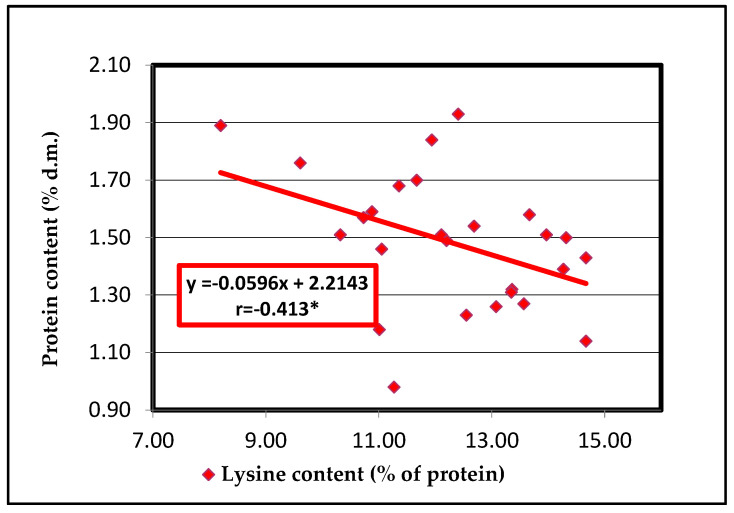
Regression line between protein and lysine contents of 26 barley seed accessions.

**Figure 9:**
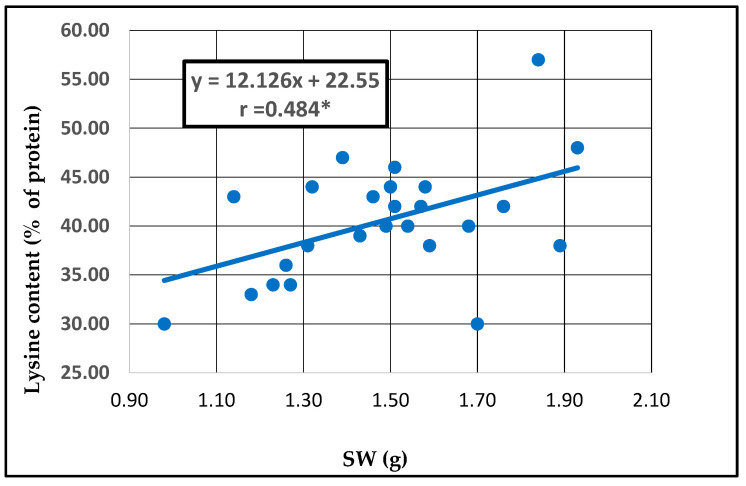
Regression line between lysine content and SW of 26 barley seed accessions.

In barley accessions, a significant negative correlation was observed between the protein and lysine contents (r = −0.413 *), and a significant positive correlation between lysine content and the SW (r = 0.484 *). Other correlations are not statistically assured. To emphasize the significant correlations, the corresponding regression lines were observed (Figure 8 and Figure 9).

In barley genotypes, an increased protein content does not lead to an increased lysine content, showing a negative relationship between protein and lysine contents. Regression lines between lysine content and the SW showed a significant correlation, which means that the level of lysine content will be higher when the barley grain has an SW up to 57 g.

### 3.3. The Comparasion of Results Based on Biochemical and Physical Markers

In order to highlight the role of these species in human nutrition, a comparative analysis of the obtained results for the studied oat and barley genotypes was carried out.

A higher protein content was observed for the barley seed accessions compared to oat. According to the studies conducted by Băișan and Baniwal in 2021 [27,28], oat grains had a protein content between 9 and 20% d.m., and barley grains between 9 and 11% d.m. In the present study, the oat protein values ranged between 7.02 and 14.23% d.m. and, in barley, between 8.2 and 14.67% d.m.

**Figure 10:**
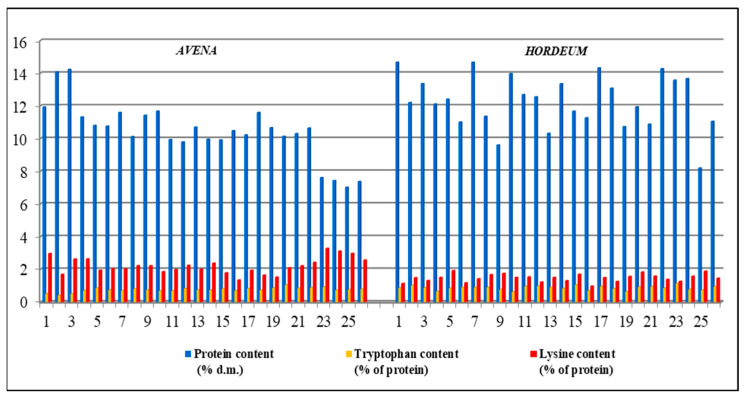
Protein, tryptophan and lysine contents of oat and barley genotypes.

Lysine content was higher in oat (1.35–3.29% of the total protein) than in barley (0.98–1.89% of the total protein); in some cases, the values of oat were 100% higher compared to those obtained for barley. The tryptophan content was higher in barley (0.62–1.14% of the total protein) than in oat (0.41–1.05% of the total protein). The lysine and tryptophan values were quite low, similar to the results reported in the literature by Biel in 2020 [26], which specified that oat and barley grains contain a large number of amino acids, but the essential ones (lysine and tryptophan), even if found in small amounts compared to the requirements of the human body, offer a good nutritional value of protein (Figure 10).

**Figure 11:**
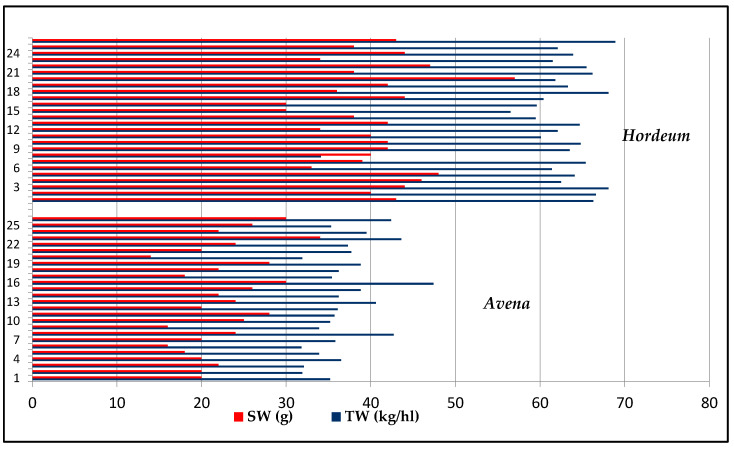
Values of TW and SW of oat and barley genotypes.

Regarding physical evaluation, both TW and SW presented higher values in barley genotypes compared to oat. In the case of the SW of oat, some values almost doubled (Figure 11).

**Figure 12:**
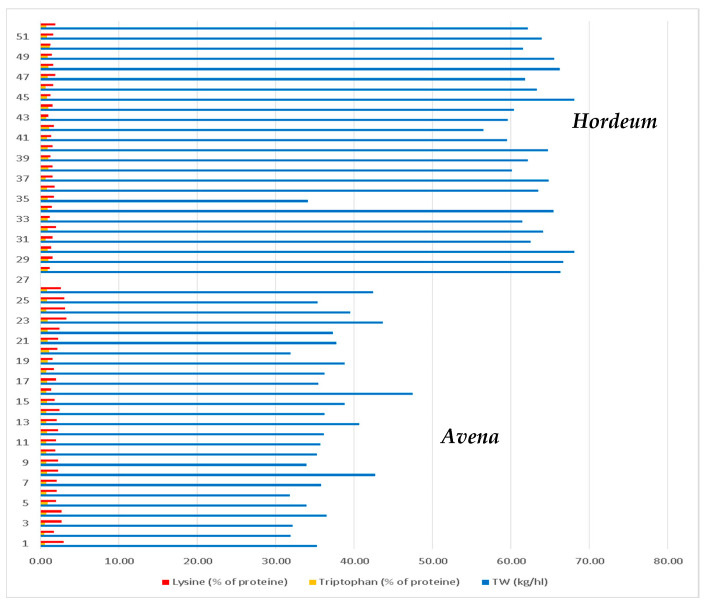
Values of lysine, tryptophan contents and TW of Oat and barley genotypes.

By summarizing the obtained results, it was observed that lysine are found in larger quantities in oat seeds, but barley seeds have a much higher TW than oat seeds (Figure 12).

Similar results were observed in other studies as well [22,28]. The obtained results confirm, again, the nutritional attributes of these species and the main reasons why we should include them in our daily menu. They are important components of a healthy diet because they are a good source of nutrients and can help in the prevention and therapy of chronic diseases [29]. For example, lower numbers of and disorders in tryptophan metabolism have been associated with hypertension and kidney disease [30], vascular inflammation and cardiovascular disease [31], inflammatory bowel disease and colorectal cancer [13,32]. Similarly, the lysine deficit was correlated with cardiovascular diseases [33], neurodegenerative diseases such as Alzheimer’s [34,35], eye health [36], renal failure or diabetes [16,37].

**Figure 13:**
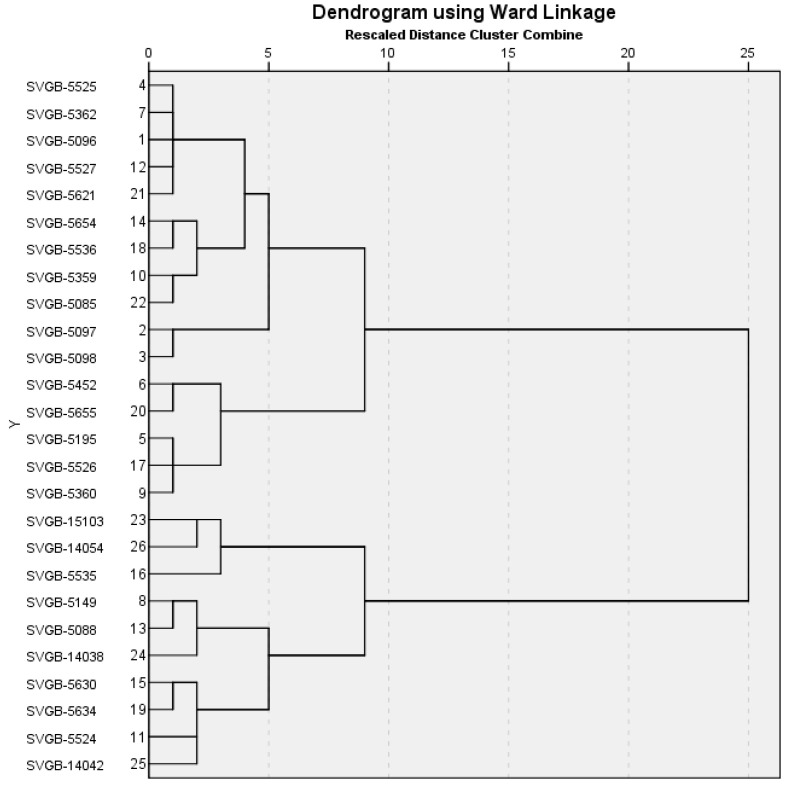
Ward’s Dendrogram, using Euclidian distances of 26 oats genotypes evaluated for protein, tryptophan and lysine contents, TW and SW.

Cluster analysis was used to group the oat genotypes into clusters based on the similarity in the performance of the physical and chemical traits included in this study. The oat genotypes were grouped into two clusters (Figure 13): 16 accessions were grouped into cluster I and 10 in cluster II. The maximum Euclidian distances were observed in two accessions: SVGB-5097 and SVGB-5098.

**Figure 14:**
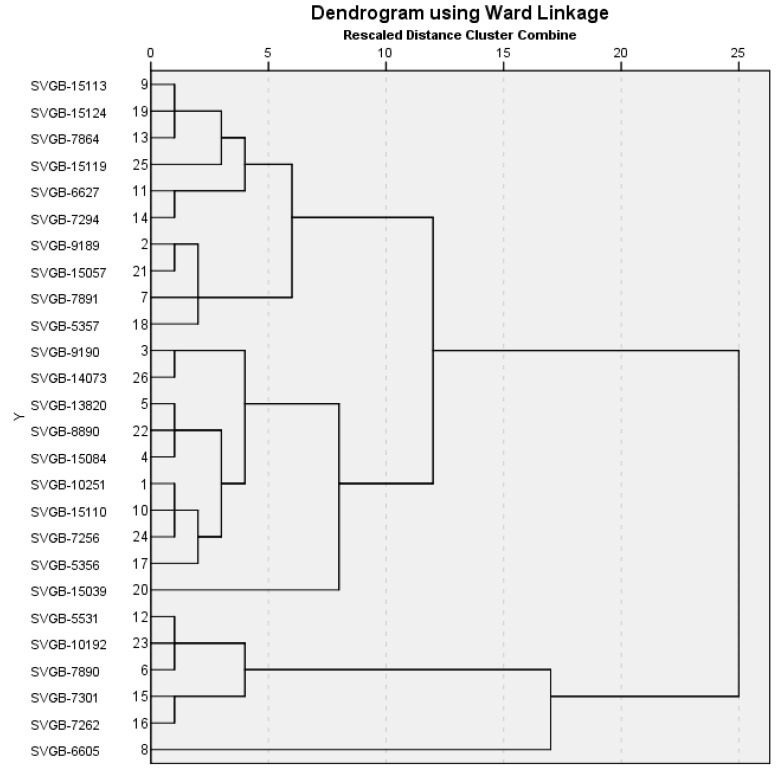
Ward’s Dendrogram, using Euclidian distances of 26 barley genotypes evaluated for protein, tryptophan and lysine contents, TW and SW.

The barley genotypes were grouped into three clusters (Figure 14): 10 accessions were grouped in cluster I, 9 in cluster II and 7 genotypes into cluster III. Ward’s Dendrogram emphasized the basis Euclidian distance, noticing maximum Euclidian distances in cluster III (SVGB-5531, SVGB-10192, SVGB-7301, SVGB-7262 and SVGB-6605).

The oats and barley genotypes which registered the highest performance in the evaluated physico-chemical (protein, tryptophan and lysine contents, TW and SW) traits could be used in improving the quality of oat and barley varieties.

## 4. Conclusions

In the current study, we carried out physico-chemical analyses on 52 genotypes of oat and barley for Suceava Gene Bank collection. The studied oat and barley accessions showed significant variability if we consider the physico-chemical properties (protein, lysine, tryptophan, TW and SW) analyzed. The highest variability was observed for protein content, but for essential amino acids (lysine, tryptophan), the amplitudes of variation have been lower. The protein content was much higher in barley genotypes analyzed compared to oat, although lysine was higher in oat than in barley, while tryptophan presented higher values in barley compared to oat accessions.

In terms of physical indicators, TW and SW showed higher values in barley genotypes compared to oats, with SW values almost double those of oats. Regarding physico-chemical properties, both species had a small to medium variability, except for the SW of oat accessions, which shows a higher variability (CV% = 21.19). Also, according to the statistical results, correlations between biochemical components were observed. In oat samples, there were two distinctly significant negative correlations between protein and lysine content (r = −0.591 **) and between protein content and TW (r = −0.502 **), and two significant negative correlations between protein and tryptophan content (r = −0.447 *) and between protein content and SW (r = −0.433 *). There was a highly significant positive correlation between SW and TW (r = 0.747 ***). A negative relationship between protein content and lysine and tryptophan, as well as between protein content and TW and SW, was noticed. These results demonstrate that the content of essential amino acids (lysine, tryptophan) does not increase with increasing protein content. Also, an increase in caryopsis size does not increase the protein content of the grain. In barley, a significant negative correlation was observed between protein and lysine contents (r = −0.413 *), which means that there is no lysine content increasing at a higher protein content.

Two positive correlations between protein and tryptophan content and between lysine content and SW (r = 0.484 *) were observed; thus, the lysine content increased with the increasing size of grain. Pearson correlation coefficients indicated that in oat, there is a strong relationship between SW, TW and lysine, and in barley, between protein, tryptophan and TW. Both results demonstrated the importance of test weight in grain quality, and also in the content of these two essential amino acids (lysine, tryptophan). Through biochemical and nutritional characterization of the genetic material studied, two oat genotypes (SVGB-5360, SVGB-5085) and two barley genotypes (SVGB-6627, SVGB-5356) were identified with above average values of the two essential amino acids (lysine, tryptophan).

For oat genotypes, the maximum Euclidean distances based on the evaluated physico-chemical characteristics were identified in two oat accessions (SVGB-5097, SVGB-5098) and for barley five accessions (SVGB-5531, SVGB-10192, SVGB-7301, SVGB-7262, SVGB-6605). These accessions could be used to improve the quality of oats and barley varieties for human consumption.

## Figures and Tables

**Table 1:** Characteristics of the oat and barley seed accessions tested.

Oat					Barley				
Accession Number	Origin	Latitude	Longitude	Altitude	Accession Number	Origin	Latitude	Longitude	Altitude
*SVGB-5096*	Polonia	-	-	-	*SVGB-10251*	Satu Mare, Agriș	475301N	0230017E	200
*SVGB-5097*	Polonia	-	-	-	*SVGB-9189*	Cluj, Băișoara	463500N	0232800E	551
*SVGB-5098*	Polonia	-	-	-	*SVGB-9190*	Cluj, Băișoara	463500N	0232800E	551
*SVGB-5525*	Alba, Scărișoara	462745N	0225301E	920	*SVGB-15084*	Sălaj, Bucium, Bogdana	470140N	0230101E	464
*SVGB-5195*	Bihor, Șuncuiuș	465721N	0222725E	550	*SVGB-13820*	Maramureș, Botiza	474009N	0240950E	480
*SVGB-5452*	Alba, Săsciori	455331N	0233322E	650	*SVGB-7890*	Covasna, Brețcu	460300N	0261800E	639
*SVGB-5362*	Hunedoara, Valea Bradului	460935N	0224946E	520	*SVGB-7891*	Covasna, Brețcu	460300N	0261800E	639
*SVGB-5149*	Russia	-	-	-	*SVGB-6605*	Pakistan	-	-	-
*SVGB-5360*	Caraș-Severin, Măgura, Zăvoi	452300N	0214800E	284	*SVGB-15113*	Cluj, Calata	464800N	0230100E	689
*SVGB-5359*	Maramureș, Moisei	473940N	0243149E	700	*SVGB-15110*	Cluj, Călățele	464600N	0230100E	744
*SVGB-5524*	Hunedoara, Valea Bradului	460935N	0224946E	520	*SVGB-6627*	Argentina	-	-	-
*SVGB-5527*	Maramureș, Botiza	474009N	0240950E	480	*SVGB-5531*	Alba, Câmpeni, Certege	462217N	0230224E	750
*SVGB-5088*	Mureș, Reghin	464628N	0244233E	400	*SVGB-7864*	Cluj, Mihai Viteazu, Cheia	463200N	0234300E	325
*SVGB-5654*	Cluj, Răchițele	464200N	0225400E	983	*SVGB-7294*	Mureș, Hodac	464633N	0245533E	450
*SVGB-5630*	Hunedoara, Ghelari, Ruda	454158N	0224701E	800	*SVGB-7301*	Mureș, Hodac	464633N	0245533E	450
*SVGB-5535*	Maramureș, Moisei	473940N	0243149E	700	*SVGB-7262*	Harghita, Sărmaș, Hodoșa	465300N	0252800E	894
*SVGB-5526*	Alba, Câmpeni, Certege	462217N	0230224E	750	*SVGB-5356*	Bistrița Năsăud, Lunca Ilvei	472200N	0245900E	715
*SVGB-5536*	Maramureș, Moisei	473940N	0243149E	700	*SVGB-5357*	Bistrița Năsăud, Lunca Ilvei	472200N	0245900E	715
*SVGB-5634*	Brașov, Sohodol	453100N	0252400E	924	*SVGB-15124*	Cluj, Mănăstireni	464600N	0230500E	812
*SVGB-5655*	Cluj, Râșca	464300N	0230600E	824	*SVGB-15039*	Sălaj, Cizer, Pleșca	470536N	0225351E	486
*SVGB-5621*	Hunedoara, Ghelari, Ruda	454158N	0224701E	800	*SVGB-15057*	Sălaj, Horoatu Crasnei, Ponița	470313N	0225432E	382
*SVGB-5085*	Bistrița Năsăud, Rodna	472500N	0244900E	601	*SVGB-8890*	Bistrița Năsăud, Rebra	471925N	0243015E	684
*SVGB-15103*	Sălaj, Hida, Păduriș	470653N	0232455E	274	*SVGB-10192*	Maramureș, Săliște	472939N	0230601E	600
*SVGB-14038*	Maramureș, Ungureni	473151N	0235946E	414	*SVGB-7256*	Harghita, Sărmaș	465300N	0252800E	894
*SVGB-14042*	Maramureș, Peteritea	472533N	0234514E	400	*SVGB-15119*	Cluj, Călățele, Văleni	464700N	0230200E	754
*SVGB-14054*	Maramureș, Vima Mică	472425N	0234303E	406	*SVGB-14073*	Maramureș, Vima Mare	472317N	0234137E	406

**Table 2:** Mean and range of protein, lysine, tryptophan contents, TW and SW of 26 oat seed accessions.

Specification	Average Value	Maximum Value	Accession Number	Minimum Value	Accession Number	Variance	CV%
Protein content (% d.m.)	10.45	14.23	SVGB-5098	7.02	SVGB-14042	3.02	16.65
Lysine content (% of protein)	2.22	3.29	SVGB-15103	1.35	SVGB-5535	0.244	10.99
Tryptophan content (% of protein)	0.75	1.05	SVGB-5655	0.41	SVGB-5097	0.017	17.60
TW (kg/hL)	37	47.4	SVGB-5535	31.8	SVGB-5452	15.173	10.51
SW (g)	22.65	34	SVGB-15103	14	SVGB-5655	23.115	21.19

**Table 3:** Correlation coefficients between protein, lysine, tryptophan contents, TW and SW of 26 oat genotypes.

Correlated Traits	Protein Content (% d.m.)	Lysine Content (% of Protein)	Tryptophan Content (% of Protein)	TW (kg/hL)	SW (g)
Protein content (% d.m.)					
Lysine content (% of protein)	−0.591 **				
Tryptophan content (% of protein)	−0.447 *	0.006			
TW (kg/hL)	−0.502 **	0.241	0.033		
SW (g)	−0.433 *	0.031	0.111	0.747 ***	

**Table 4:** Mean and range of protein, lysine, tryptophan contents, TW and SW of 26 barley seed accessions.

Specification	Average Value	Maximum Value	Accession Number	Minimum Value	Accession Number	Variance	CV%
Protein content (% d.m.)	12.26	14.67	SVGB-10251 SVGB-7891	8.2	SVGB-15119	2.66	13.29
Lysine content (% of protein)	1.48	1.89	SVGB-15119	0.98	SVGB-7262	0.05	15.81
Tryptophan content (% of protein)	0.86	1.14	SVGB-10192	0.62	SVGB-15110	0.01	14.88
TW (kg/hL)	62.3	68.9	SVGB-14073	34.1	SVGB-6605	42.19	10.42
SW (g)	40.54	57	SVGB-15039	30	SVGB-7301 SVGB-7262	34.49	14.48

**Table 5:** Correlation coefficients between protein, lysine, tryptophan contents, TW and SW of 26 barley genotypes.

Correlated Traits	Protein Content (% d.m.)	Tryptophan Content (% of Protein)	Lysine Content (% of Protein)	TW (kg/hL)	SW (g)
Protein content (% d.m.)					
Tryptophan content (% of protein)	0.183				
Lysine content (% of protein)	−0.413 *	−0.066			
TW (kg/hL)	0.162	−0.088	−0.188		
SW (g)	0.166	0.203	0.484 *	0.174	

## Data Availability

The original contributions presented in this study are included in the article; further inquiries can be directed to the corresponding author.
